# Analysis of a Mouse Skin Model of Tuberous Sclerosis Complex

**DOI:** 10.1371/journal.pone.0167384

**Published:** 2016-12-01

**Authors:** Yanan Guo, John R. Dreier, Juxiang Cao, Heng Du, Scott R. Granter, David J. Kwiatkowski

**Affiliations:** Brigham and Women’s Hospital, Harvard Medical School, Boston, Massachusetts, United States of America; Texas A&M University Health Sciences Center, UNITED STATES

## Abstract

Tuberous Sclerosis Complex (TSC) is an autosomal dominant tumor suppressor gene syndrome in which patients develop several types of tumors, including facial angiofibroma, subungual fibroma, Shagreen patch, angiomyolipomas, and lymphangioleiomyomatosis. It is due to inactivating mutations in *TSC1* or *TSC2*. We sought to generate a mouse model of one or more of these tumor types by targeting deletion of the *Tsc1* gene to fibroblasts using the *Fsp-Cre* allele. Mutant, *Tsc1*^*cc*^*Fsp-Cre+* mice survived a median of nearly a year, and developed tumors in multiple sites but did not develop angiomyolipoma or lymphangioleiomyomatosis. They did develop a prominent skin phenotype with marked thickening of the dermis with accumulation of mast cells, that was minimally responsive to systemic rapamycin therapy, and was quite different from the pathology seen in human TSC skin lesions. Recombination and loss of Tsc1 was demonstrated in skin fibroblasts in vivo and in cultured skin fibroblasts. Loss of Tsc1 in fibroblasts in mice does not lead to a model of angiomyolipoma or lymphangioleiomyomatosis.

## Introduction

Tuberous Sclerosis Complex (TSC) is an autosomal dominant genetic disorder due to inactivating mutations in either *TSC1* or *TSC2*. There are well-established consensus diagnostic criteria for TSC; the major clinical criteria include angiofibromas or fibrous cephalic plaque, ungual fibromas, shagreen patch, angiomyolipomas, and lymphangioleiomyomatosis [1]. Tumors that develop in TSC are known to develop through a two hit mechanism, in which there is uniform germ-line or mosaic inactivating mutation in *TSC1* or *TSC2*, and second hit inactivating mutation in the other allele of the same gene to lead to loss of function of either TSC1 or TSC2 in the cells comprising the tumor [2–8]. Similar findings have been made in the majority of sporadic angiomyolipoma and lymphangioleiomyomatosis lesions [3–5, 8].

Heretofore no mouse model has been generated which replicates the core features of any of these TSC tumors, including angiofibromas, ungual fibromas, shagreen patch, angiomyolipomas, or lymphangioleiomyomatosis. Here we sought to generate such a model by targeting bi-allelic deletion of *Tsc1* to fibroblasts. Fibroblasts or a closely related cell type are thought to be the cell which gives rise to angiofibromas, ungual fibromas, and shagreen patch [2], and might be the cell which gives rise to angiomyolipoma and lymphangioleiomyomatosis.

## Materials and Methods

### Mice and drug treatments

All procedures were carried out in accordance with the Guide for the Humane Use and Care of Laboratory Animals, and the study was approved by the Animal Care and Use Committee of Children's Hospital, Boston. The approved protocol contained the following procedures. Euthanasia was performed using CO2 narcosis. Mice were monitored 3 times per week. If any of the following conditions occurred mice were subject to euthanasia: skin irritation or breakdown, tumors > 10% body weight, tumors that limit mobility, lethargy, loss of appetite, weight loss > 15–20%, Rodent Body Condition Score of 1 or 2. Some of the *Tsc1*^*cc*^*Fsp-cre+* mice were subject to euthanasia for these reasons. None died spontaneously. Some mice were treated with Baytril cream for dermatitis to alleviate suffering, but anesthetics, analgesics, or other treatments were not used. Mice were housed under standard conditions meeting the standards of the Guide for the Humane Use and Care of Laboratory Animals, with strict cage crowding limits, a temperature of 70°F and a 12 hour light/dark cycle.

*Tsc1* conditional allele mice, denoted *Tsc1*^*cc*^ were originally derived in this laboratory [9] (^c^ denotes conditional, floxed allele; ^w^ denotes wild type allele). Mice with the *Fsp-Cre* allele were a generous gift of Gustavo Leone, Ohio State University, and were described in [10]. In these mice, Cre expression is driven by a 3.1-kb gene promoter sequence extending from 1.9 kb upstream of exon 1 to the end of intron 1 of the *S100A4* gene (formerly known as Fibroblast specific protein1, or Fsp1). Mice were derived by standard breeding strategies. *Tsc1*^*cc*^*Fsp-cre+* mice were compared to *Tsc1*^*cw*^*Fsp-cre+*, *Tsc1*^*cc*^, and *Tsc1*^*cw*^ mice.

DNA was prepared from mouse toes/tails by standard procedures for genotyping. PCR genotyping for *Tsc1* was performed using a three-primer system that allows simultaneous analysis of wildtype (w), conditional (c), and knockout (k) alleles, followed by agarose gel electrophoresis [9]. Primers that amplify a 300 bp portion of the cre recombinase were used to assess the presence of the *Fsp-cre* allele.

Rapamycin was purchased from LC laboratories (Woburn, MA). A 20 mg/ml stock was made using ethanol, and mixed daily for injection with sterile vehicle (0.25% PEG-200, 0.25% Tween-80). Mice were treated with rapamycin by intraperitoneal (IP) injection at 3 mg/kg three times per week for four weeks. Control mice received the vehicle solution IP on the same schedule.

### Pathology studies

Six micrometer sections were prepared from tissues fixed with 10% formalin overnight followed by 70% ethanol and paraffin embedding, and stained with hematoxylin and eosin by standard techniques at the Rodent Pathology Core at Harvard Medical School.

Skin samples were collected from both ventral and dorsal mouse skin. Skin thickness was quantified by a dermatopathologist (SG) who was blinded to the genotype of the mice. Multiple regions across a single long biopsy of skin were examined and measured at three distinct sites, and averaged to determine the skin epidermal and dermal thickness for each mouse. Chloroacetate esterase staining of skin sections was performed to identify mast cells. Sections were deparaffinized with xylene, put through an alcohol series, treated with Napthol AS-D chloroacetate esterase (Sigma) and counterstained with hematoxylin. Mast cells per high powered field (400x) were quantified in a blinded manner by a dermatopathologist (SG).

For immunofluorescence studies, ventral skin samples were flash frozen in OCT, cut in 5 micron sections, blocked in PBS with 10% milk, and incubated with anti-pS6 (S235/S236) (CST, at 1:100 dilution) and anti-Cre (CST, at 1:800 dilution) antibodies. Secondary antibodies were also obtained from CST, and were conjugated with Alexa-488 and Alexa-555 fluorophore (used at 1:500 dilution). Slides were mounted using ProLong Gold Antifade with DAPI (cat# P36941, Thermo). Sections were viewed using a Fluoview FV10i (Olympus) confocal microscope and analyzed using ImageJ (National Institutes of Health) software.

### Primary fibroblast cell culture and immunoblotting

Fibroblast cell lines were derived from the skin of mouse pups of age P0-P7 from a breeding of *Tsc1*^*cc*^*Fsp-cre+* x *Tsc1*^*cc*^ mice. After euthanasia, and 70% ethanol sterilization, skin samples (1cm x 1cm) were collected from the underarm or abdominal areas. Skin samples were washed again in 70% ethanol and then washed in PBS. They were cut into small pieces, treated with 0.2% collagenase (Sigma) in serum-free Ham’s F-12 media (Invitrogen) at 37°C for 1hr, with agitation every 20 min. The cells were washed with PBS 3 times, plated in fibroblast culture medium, and maintained in an incubator at 37°C with 5% CO_2_. Fibroblast culture medium is a 1:1 mix of DMEM and F-12 (Invitrogen) containing 15% FBS (Invitrogen), 1% HEPES buffer solution (GIBCO), 1% nonessential amino acid mixture (GIBCO), 1% L-glutamine (GIBCO), 1% penicillin/streptomycin (Cellgro) and 1% sodium pyruvate (GIBCO). For serum starvation, cells were cultured in the absence of serum for 24 h.

Cells were harvested in lysis buffer consisting of 50 mM Tris-HCl (pH 7.5), 150 mM NaCl, 1% Triton X-100, 1 mM EDTA, 1 mM EGTA and a cocktail of protease inhibitors (Sigma-Aldrich, St. Louis, MO). Cell lysates were clarified by centrifugation for 5 min at 14k rpm, and the protein concentration of the supernatants was determined using a modified Bradford assay (Bio-Rad, Hercules, CA). For immunoblotting, 10 μg of protein was loaded in each lane, and was separated by SDS-PAGE on 4–12% gradient gels (Invitrogen, Carlsbad, California), transferred to PVDF membranes and detected by immunoblotting with the following primary antibodies: TSC1, TSC2, pAKT(S473), AKT, pS6(S240/244), S6, pS6K1(Thr389), pS6K1, and actin (all from Cell Signaling Technology; all used at 1:1,000 dilution, except for actin, used at 1:15,000). Goat anti-mouse and anti-rabbit secondary antibodies (Santa Cruz Biotechnology, Santa Cruz, CA) conjugated to horseradish peroxidase were used at a 1:3000 dilution and immunoreactive bands were detected by chemiluminescence (SuperSignal, Pierce, Rockford, IL) and film (Denville Scientific, South Plainfield, NJ).

### Statistical analysis

All data points are shown in relevant figures as dot plots. Average values are indicated by a horizontal line. P values were calculated using GraphPad Prism v7.0a using the Mann-Whitney test.

## Results

### *Fsp-cre* mediated loss of *Tsc1* leads to multiple phenotypes including premature mortality

**Fig 1:**
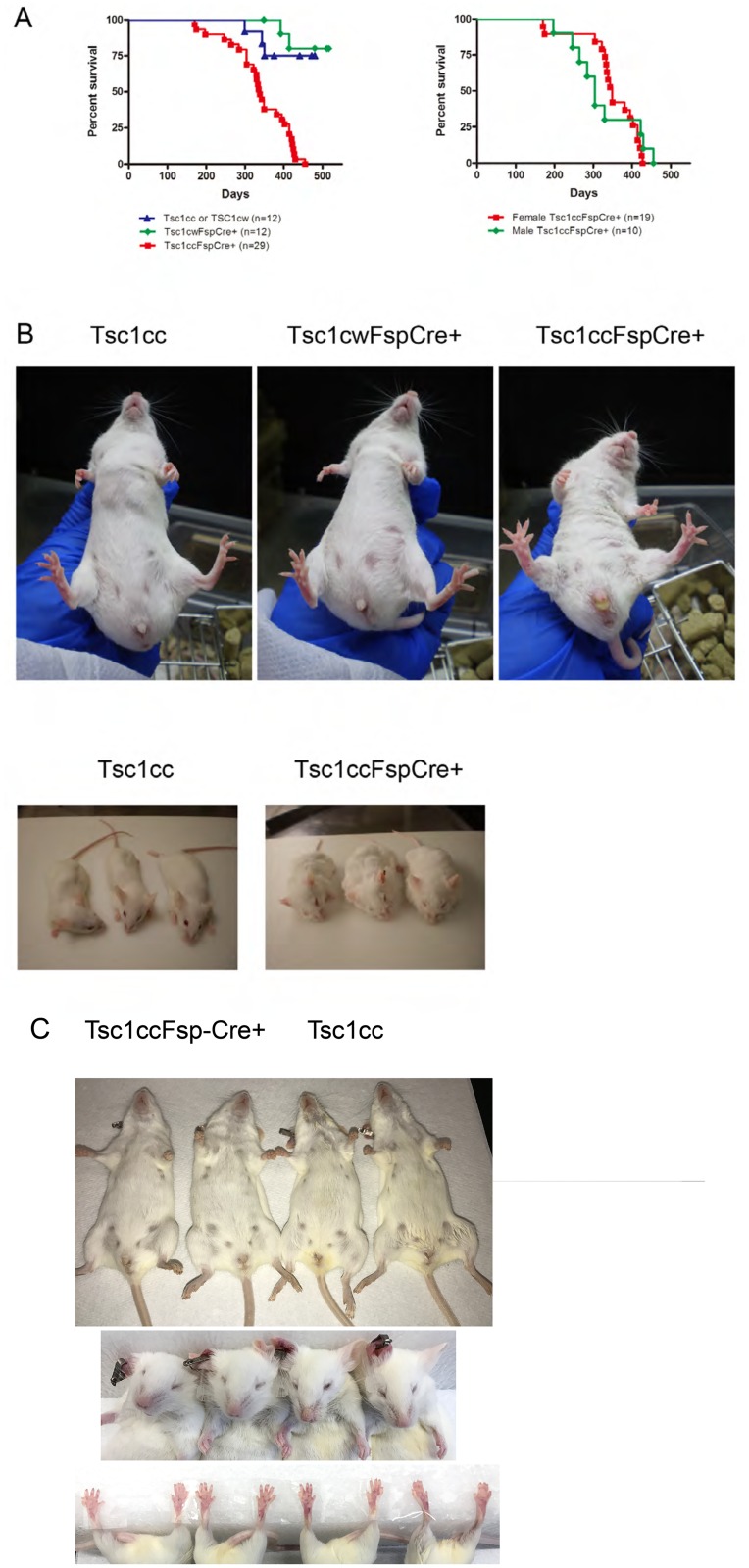
Survival and gross skin features of *Tsc1*^*cc*^*Fsp-cre+* mice. A. Survival curve of mice of various genotypes. Note reduced survival of *Tsc1*^*cc*^*Fsp-cre+* mice in comparison to other genotypes (p<0.0001). The median survival of *Tsc1*^*cc*^*Fsp-cre+* mice is 338 days. Right, there was no major difference between the survival of female and male *Tsc1*^*cc*^*Fsp-cre+* mice (p = 0.2660). The median survival of females and males is 349 days and 304 days respectively. B. Pictures of mice of various genotypes are shown. Note the loose, somewhat wrinkled skin seen in the *Tsc1*^*cc*^*Fsp-cre+* mice in comparison to other genotypes. All mice shown had age 8 months. C. Additional pictures of mice at age 1 year, with two *Tsc1*^*cc*^*Fsp-cre+* mice on left and two *Tsc1*^*cc*^ mice on right. Note wrinkled loose skin on abdomen, blunt nose, and larger paws of the *Tsc1*^*cc*^*Fsp-cre+* mice in comparison to the *Tsc1*^*cc*^ mice.

To attempt to develop a mouse model of Shagreen patch, cephalic patch, facial angiofibroma, ungual fibroma, renal angiomyolipoma, and/or lymphangioleiomyomatosis, we used an *Fsp-cre* transgene [10] to drive loss of *Tsc1* in fibroblasts in mice in vivo. Through initial breeding experiments we found that *Tsc1*^*cc*^*Fsp-cre+* mice were generated at Mendelian ratios, displayed no unusual mortality through the age of 6 months, and that both sexes were fertile. However, *Tsc1*^*cc*^*Fsp-cre+* mice began to die at 6 months and had a median survival of 338 days in contrast to *Tsc1*^*cw*^*Fsp-cre+* and *Tsc1*^*cc*^ littermates (Fig 1A, p < 0.0001). Male and female *Tsc1*^*cc*^*Fsp-cre+* mice showed a similar reduction in survival (Fig 1A right). However, female *Tsc1*^*cc*^*Fsp-cre+* mice all died due to the development of chylous ascites, while male *Tsc1*^*cc*^*Fsp-cre+* mice died of sudden death. *Tsc1*^*cc*^*Fsp-cre+* mice (n = 12; 4 males, 8 females; age 9–14 months) displayed multiple findings at necropsy including osteopetrosis (100%), liver hemangioma (67%), hyperplastic vessels (33%) and uterine rhabdomyosarcoma (88% in females). Other findings included stomach adenoma, lung adenoma, lung thrombus, thymic atrophy, ear angioma, and aortic angioma (intraluminal). However, there was no evidence of angiomyolipoma in the kidneys or other organs of these mice. Nor were any pulmonary lesions or cystic lung disease identified.

Hence we focused on the skin manifestations seen in the *Tsc1*^*cc*^*Fsp-cre+* mice.

### Tsc1^cc^FspCre+ mice have a major skin phenotype

A clear and universal phenotype observed in the *Tsc1*^*cc*^*Fsp-cre+* mice was the development of loose, apparently excessive skin on both the dorsal and ventral surfaces (Fig 1B and 1C). This appeared by one month of age, and was a reliable marker of genotype in all mice. *Tsc1*^*cc*^*Fsp-cre+* mice also had a blunt nose, reduced eye opening, paw enlargement (Fig 1C), and dermatitis, as reflected in repetitive scratching behavior. Repetitive scratching often led to dermatitis, and a need to sacrifice the mouse on a humane basis. Interestingly, this was seen only in male (19 of 25, 76%) and not in female (0 of 13) mice (p<0.0001, Fisher’s exact test).

**Fig 2:**
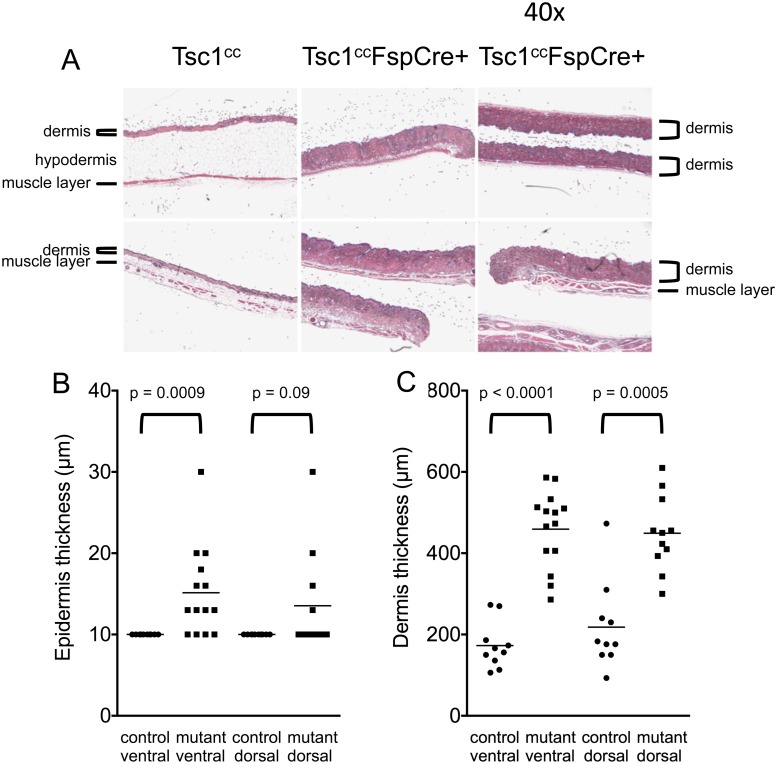
Skin histology in *Tsc1*^*cc*^*Fsp-cre+* mice. A. Representative skin H&E sections are shown for mice of each genotype at 1 year of age. All images shown are taken at 40x magnification. Top panel is dorsal and the bottom panel is ventral skin. Several layers are indicated in selected images, including dermis, hypodermis, and muscle layers. Note the increase in the dermal thickness and marked reduction in the hypodermis in *Tsc1*^*cc*^*Fsp-cre+* mice compared to *Tsc1*^*cc*^ mice. B, C. Quantitation of epidermis (B) and dermis (C) thickness is shown for control (solid circles) and Tsc1ccFsp-cre+ (solid squares) mice. Note the increase in both epidermal and dermal thickness, which is statistically significant in all cases except control vs. mutant dorsal epidermis. n = 10–14 for each measurement; mice had ages 8–13 months.

**Fig 3:**
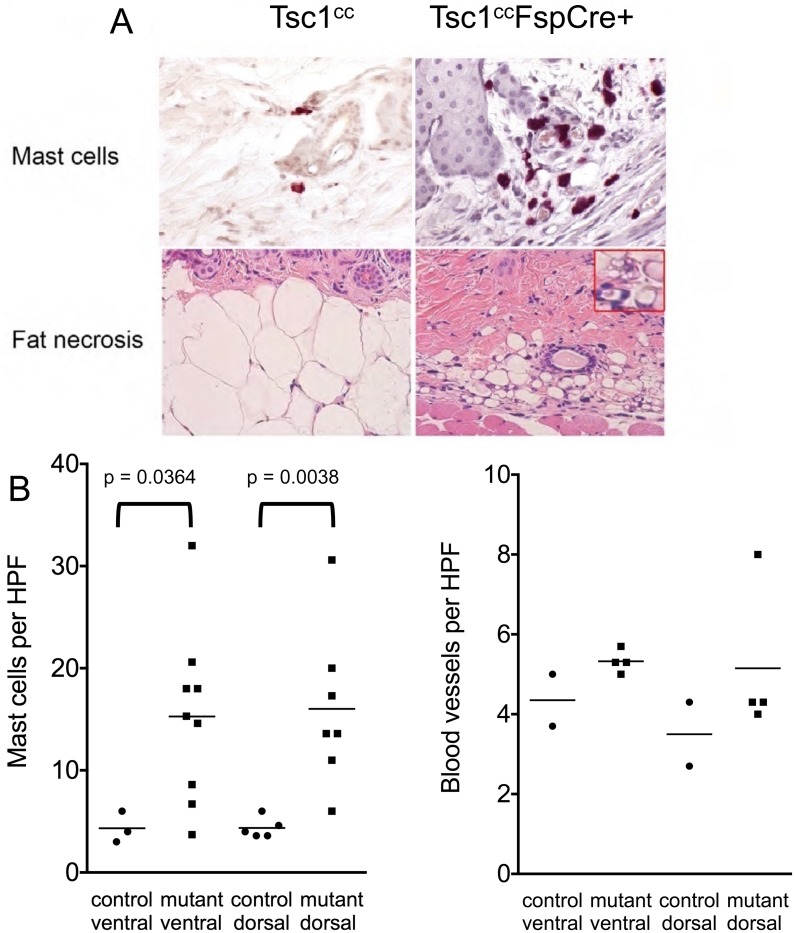
Increased mast cells and fat necrosis are seen in the skin of *Tsc1*^*cc*^*Fsp-cre+* mice. A. High power images (400x magnification) of *Tsc1cc* and *Tsc1ccFsp-cre+* mouse skin at age 1 year is shown. Upper panel, chloroacetate esterase stain to highlight mast cells. Bottom panel, H&E stain showing fat necrosis, inset at 1000x. B. Quantification of mast cells per high-powered field (400x) as assessed by dermatopathologist (SG). Mast cells are significantly increased in both dorsal and ventral skin of Tsc1^cc^FspCre1+ mice compared to Tsc1cc mice. n = 3–9 for each measurement; mice had ages 8–13 months. C. Quantification of blood vessel density per high-powered field (400x) as assessed by dermatopathologist (SG). There is a possible trend toward more vessels in the mutant mouse skin. n = 2–4 for each measurement; mice had ages 8–13 months.

Normal mouse skin consists of a very thin surface epithelium (epidermis), a thicker connective tissue layer (dermis), and a much larger adipose tissue layer (hypodermis) (Fig 2A left). A thin layer of striated muscle separates the skin from other structures. *Tsc1*^*cc*^*Fsp-cre+* mice displayed an approximate two-fold increase in the thickness of the dermis (Fig 2C, p < 0.0001 for ventral dermis, p = 0.0005 for dorsal dermis) coupled with a more modest increase in ventral and dorsal epidermis thickness (Fig 2B, p = 0.0009 for ventral epidermis, p = 0.09 for dorsal epidermis). Many sections also showed a marked reduction in the hypodermis or adipose layer. Inspection of H & E sections suggested that there was an increased number of mast cells in the dorsal dermal layer, and chloroacetate esterase staining was performed to enable enumeration of mast cells (Fig 3A). There was an average 3.6-fold increase in mast cells per HPF in the mutants (Fig 3B) in both dorsal (p = 0.0038) and ventral (p = 0.036) skin. This was not due to repetitive scratching behavior, since that occurred only on the dorsal skin.

Given these abnormal features of the skin in the *Tsc1*^*cc*^*Fsp-cre+* mice, we hypothesized that there would be a concordant increase in blood vessel density, as seen in both facial angiofibromas and angiomyolipomas in TSC. However, using endomucin immunohistochemistry to identify blood vessels, we found that blood vessel density was not significantly different between normal and mutant skin on either dorsal or ventral sites, though there was a trend toward more blood vessels in the mutant mice (Fig 3C).

As noted above, the hypodermis layer was markedly thinned in the *Tsc1*^*cc*^*Fsp-cre+* mice (Fig 2A). Fat necrosis was seen consistently in the mutant mice in this region, and was likely a major contributor to hypodermal atrophy (Fig 3A bottom row).

### Analysis of recombination and mTORC1 activation in *Tsc1*^*cc*^*Fsp-cre+* mice skin

**Fig 4:**
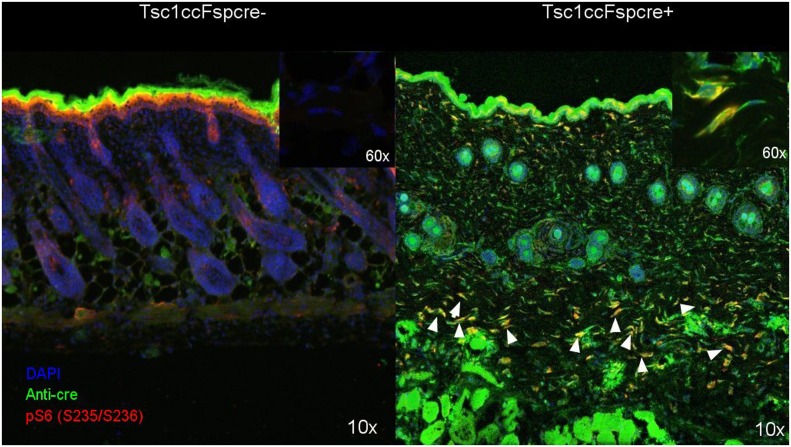
Identification of Cre expression and mTORC1 activation in the skin of *Tsc1ccFsp-cre+* mice. Confocal microscopy fused images (100x) are shown with nuclear (DAPI, blue), Cre (Anti-Cre antibody, green), and pS6(S235/236) (antibody, red) are shown. Multiple cells with appearance of fibroblasts are seen in the lower dermis of the mutant *Tsc1*^*cc*^*Fsp-cre+* mouse skin, that stain with both red and green stains (yellow, indicated by arrow heads). Some of these cells are shown at higher magnification in the inset in the upper right corner (600x). A similar region of the control mouse skin shows no staining.

The *Fsp-cre* transgene has been reported to lead to high level recombination in mesenchymal lineage cells including fibroblasts [10]. To define the extent of cre expression and recombination, we performed confocal microscopy of fresh-frozen OCT-embedded skin sections of the mice, using anti-Cre and anti-pS6(S235/S236) antibodies. Although there was considerable background staining by the anti-Cre antibody, a population of Cre+ pS6+ fibroblast cells were clearly identified in the Dermis layer of skin of the *Tsc1*^*cc*^*Fsp-cre+* mice, and not seen in control skin sections (Fig 4). This indicates that dermal fibroblasts are showing cre expression and activation of mTORC1, as expected.

### Rapamycin treatment reduces dermal thickness and mast cell numbers but does not rescue skin abnormalities to a major extent

**Fig 5:**
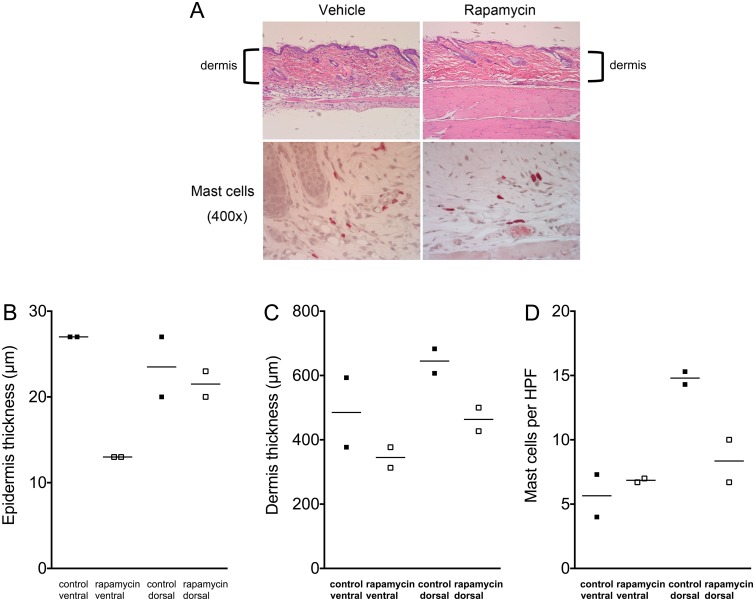
Response of the skin of *Tsc1*^*cc*^*Fsp-cre+* mice to one month treatment with rapamycin. A. Low and high power images of *Tsc1ccFsp-cre+* mouse skin at age 1 year is shown. Upper panel, H&E stain, with dermis indicated. Lower panel, chloroacetate esterase stain highlights mast cells. B-D. Quantification of epidermis and dermis thickness, and mast cell count per high-powered field (400x) as assessed by dermatopathologist (SG).

Rapamycin is an allosteric inhibitor of mTORC1 that markedly reduces its kinase activity for many substrates, particularly S6Kinase. It has been shown to be remarkably effective in the treatment of a variety of TSC mouse tumor and brain models, and for a number of TSC-related tumors [11–14]. To examine the potential benefit of rapamycin for this model, we performed a pilot experiment, treating *Tsc1*^*cc*^*Fsp-cre+* mice with either vehicle (n = 2) or rapamycin (n = 2) at 3mg/kg given by intraperitoneal injection 3 days per week for 4 weeks, beginning at age 3 months. Analysis of this pilot data indicated that there was a trend toward reduction of ventral epidermal thickness in treated mice, and toward reduction in mast cell count in dorsal sections (Fig 5). However, other measures appeared to be unaffected by treatment in this limited number of samples.

### *Tsc1*^*cc*^*Fsp-cre+* skin fibroblasts show Tsc1 loss and mTORC1 activation

**Fig 6:**
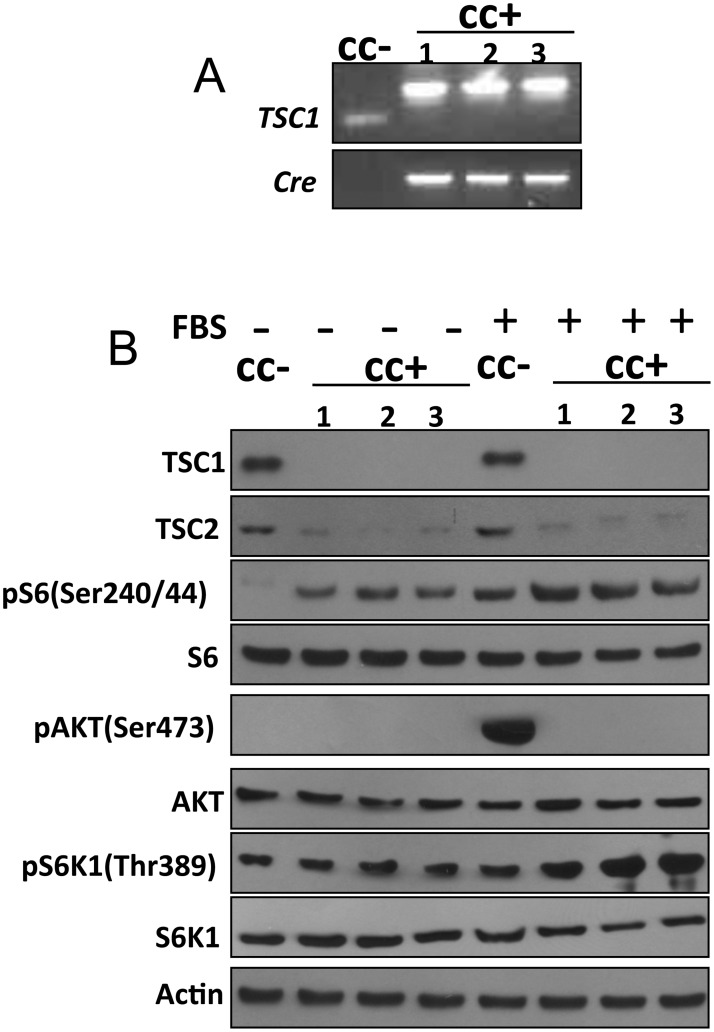
Analysis of fibroblast cell lines generated from the skin of *Tsc1*^*cc*^*Fsp-cre+* mice. A. PCR genotyping analysis of 4 fibroblast cell lines derived from littermate newborn pups. The *Tsc1*^*cc*^ cell line (cc-) has no Cre product and shows only the *Tsc1* conditional allele PCR product; the other three *Tsc1*^*cc*^*Fsp-cre+* cell lines (cc+) have both Cre PCR products and *Tsc1* knock-out (k) allele products. B. Immunoblot analysis of 4 fibroblast cell lines after 24 h of serum (FBS) starvation (-) or 30 min after serum addback following serum starvation (+). Note absent Tsc1 expression and markedly reduced Tsc2 expression by the cc+ lines; increased pS6 and pS6K1 expression and absent pAKT expression by the cc+ cell lines.

To confirm that recombination and loss of Tsc1 expression was occurring in skin fibroblasts of these mice, we prepared primary skin fibroblast cultures from *Tsc1*^*cc*^*Fsp-cre+* and control *Tsc1*^*cc*^ mice. Genotyping confirmed the presence of the *Fsp-cre* allele in these cultures, and showed that there was near complete conversion of the conditional allele to the null allele (Fig 6A). The *Tsc1*^*cc*^*Fsp-cre+* fibroblast cultures showed marked reduction of Tsc1 and Tsc2 expression, as expected. *Tsc1*^*cc*^*Fsp-cre+* fibroblasts also showed constitutive activation of mTORC1 during serum deprivation with high levels of pS6(S240/244) and some increase in pS6K(T389), and a dramatic reduction in pAKT(S473) levels in response to serum stimulation, consistent with previous data on signaling effects of mTORC1 activation in cells lacking Tsc1 (Fig 6B) [15–17].

## Discussion

Consistent with multiple previous reports [10, 18, 19], the *Fsp-cre* allele used here led to targeting of Cre recombinase expression to fibroblasts leading to recombination and loss of *Tsc1* expression in the skin fibroblasts of the *Tsc1*^*cc*^*Fsp-cre+* mice (Figs 5 and 6), and likely other tissues. The mice displayed a modest phenotype for several months until a variety of tumors developed in the male mice, and chylous ascites developed in female mice, leading to a median survival of a little less than one year. The major phenotype that was seen in these mice was in the development of the skin disorder described.

All organs of these mice were examined pathologically, with special emphasis on the kidneys and lungs in a cohort of *Tsc1*^*cc*^*Fsp-cre+* mice at ages 9–12 months. No evidence of kidney angiomyolipoma or lymphangioleiomyomatosis was seen in any mouse. These observations strongly suggest that stromal fibroblasts are not the cell of origin of these two tumors, which are known to be closely related in terms of their genetics and pathologic features [4, 5, 20–22]. Expression of melanocyte proteins, VEGFD, and Cathepsin K by these tumors suggests the possibility that the cell of origin is derived from the neural crest [23–27]. The lack of angiomyolipoma and lymphangioleiomyomatosis development in these mice are consistent with that hypothesis, as well as other possibilities.

The skin abnormalities seen in all *Tsc1*^*cc*^*Fsp-cre+* mice have are not a close match to the angiofibromas and ungual fibromas seen in TSC patients. *Tsc1*^*cc*^*Fsp-cre+* mice were shown to have thickening of the dermis with increased numbers of fibroblasts. However, there is not a prominent vascular component nor a marked increase in mast cells reported in human TSC patient lesions [28]. Furthermore, there was a minimal response to rapamycin treatment in contrast to what has been reported for TSC patients using topical rapamycin for skin treatment [29]. This may be due in part to the differences in human vs. mouse skin components, especially the abundant hair follicles present in mouse skin. In addition, however, although it can be predicted that dermal fibroblasts will undergo spontaneous loss of the second allele of *TSC1*/*TSC2* at multiple sites, the distribution of angiofibromas in TSC subjects is highly circumscribed, suggesting that other factors influence the development of these lesions beyond loss of TSC1 or TSC2. Further investigation is required.
